# On the Improbability of Measles Being Influenced by Vaccination

**Published:** 1814-04

**Authors:** Edward Rigby

**Affiliations:** Norwich


### *For the Medical and Physical Journal.*

### *On the Improbability of Measles being influenced by Vaccination; by* Mr. E. Rigby.

I LAMENT every circumstance which is likely to retard the progress of vaccination, and cannot help thinking that Dr. Watt's suggestion that the mortality from measles subsequent to vaccination is greater than after small-pox, has been too hastily brought before the public. It can, however, only be answered by a reference to facts, and there is an unfortunate difficulty in obtaining them, diseases being noticed in the bills of mortality of but few places.

The only record which I have on the subject does not favor the supposition. Scarcely any of the lower classes in this city were vaccinated before the year 1812. In 1808 and 1809 the small-pox prevailed very much, and I think it likely that fourteen hundred individuals, chiefly children, had the disease within that period, for more than two hundred died of it.* In 1810 the measles appeared, and spread very generally in the city, and the mortality from it became greater than had been known for many years; and yet it must be probable that a great proportion of the children who were infected, had the small-pox in 1808 and 1809.

Had a general vaccination taken place previously to 1808, it may be presumed there would have been two hundred more children exposed to the infection of measles in 1810, and consequently out of such an increased number, there would have been a proportional increase of deaths; for had the two hundred who were sacrificed to small-pox in 1808 and 1809 been previously vaccinated, they, probably, would have been alive in 1810, and within the influence of the infection of the disease then prevailing. On the same principle, it is equally evident that in all places where vaccination has been extensively practised, there must necessarily be a greater number to take measles, than had the population suffered its accustomed loss from small-pox.

The facts of the prevalence and mortality of the small-pox in 1808 and 1809, and of the unusual fatality of measles in 1810, I have before noticed in my *Further Facts relating to the Care of the Poor in the City of Norwich*, at pages 86, 87, and 93. EDWARD RIGBY.

*Norwich, Feb.* 20, 1814.

* On a former occasion, in my Address to the Court of Guardians, I considered the number to be twelve hundred, supposing one in six to die of small-pox; but I believe the average of the deaths is now thought to be one in seven.

